# Supplementary material for: Parvovirus minute virus of mice interacts with sites of cellular DNA damage to establish and amplify its lytic infection
Source: eLife. 2018 Jul 20;7:e37750. doi: 10.7554/eLife.37750 (PMC6095691; doi:10.7554/eLife.37750)
Supplement: Supplementary file 1. [file elife-37750-supp1.docx]

**Supplementary File 1*: Table of PCR primers and Taqman probes used in the study***

| **Primer name** | **Primer sequence** |
| --- | --- |
| **V3C-seq inverse PCR primers** | |
| MVM P4-P38 4C inverse HindIII | GAGGCAAGGTTGGTCACTACTT |
| MVM P4-P38 4C inverse NlaIII | CAGGAACTTTGCCCCATTTA |
| MVM P4-P38 4C nested inverse HindIII | GGCAAGGTTGGTCACTACTTTT |
| MVM P4-P38 4C nested inverse NlaIII | GCCCCATTTAGCACAGTAGC |
| **Primers for Taqman-qPCR analysis** | |
| MVM P4-P38 oligo for Taqman | TGCTGATCAACGCTTTATTGA |
| Chr9 CRISPR break (9qE1) oligo for Taqman | CCAAGCAATACAGACATTTGACA |
| Mouse *Ercc3* oligo for Taqman | GCCCTCCCTGAAAATAAGGA |
| Human *Ercc3* oligo for Taqman | GTAAGCAGGCTGGAGCTGAG |
| **Primers for 3C-qPCR and ChIP-qPCR Validation** | |
| Chr10 VAD (10qC1) site for 3C | ACTCAGGAGGCCTTCAATCA |
| Chr19 VAD (19qA) site for 3C | ACATTGTGGCTCACCATCTG |
| Chr15 VAD (15qE1) site for 3C | CTCCAGACAGGAGCATGAGG |
| Chr17 VAD (17qA3.3) site for 3C | ACCTTGAACTCCTGCCACAG |
| Chr17 no-VAD (17qE1.1) site for 3C | TGGCCAACACCTACTGAACA |
| Chr19 VAD (19qA) site for ChIP-qPCR (F) | ACTTCCTGGGTTCCGAGACT |
| Chr19 VAD (19qA) site for ChIP-qPCR (R) | CACAAGTGTACGAGGGCTGA |
| FRA3B for 3C | ATTCACAGTGGGGCTTACCA |
| FRA5H for 3C | GCCTCTGCTCACCAGAAGTA |
| FRA11F for 3C | TGATTCTCCTAAGCCCCTCA |
| Chr9 Cas9 break (9qE1); 3C and ChIP-qPCR | AATTGCTGAAGACACACTCTCTCTC |
| Chr9 Cas9 break (9qE1); ChIP-qPCR | AGATAACATCTTCCAGGAGTATCAAAA |
| Mouse *Ercc3* primer for nearest neighbor | GACTTCTCACCTGGGCCTACA |
| Human *Ercc3* primer for nearest neighbor | GGAAGGATCTCTGTTTAATGGAAA |
| **FISH probe PCR primers** | |
| Chr19qA VAD (F) | TCGGGAGATCATAGCTGCTT |
| Chr19qA VAD (R) | GGTCCAAGAGCAGAACTTCG |
| Chr15qE VAD (F) | CCTGCATTTTGATCCAGTCA |
| Chr15qE VAD (R) | TCTTACAGCTGCCCCTGTCT |
| Chr6pA no VAD (F) | TCAGAGACATCCCTGCTCCT |
| Chr6pA no VAD (R) | AAAACACCGTATGCCTCCAG |
| Chr17qA2 no VAD (F) | AGCTGGTGATAGCCCTGAGA |
| Chr17qA2 no VAD (R) | AGGCTAGGCACCACTCAAGA |
| Chr12qA3 no VAD (F) | GCATTCCTAGTTGGGGATCA |
| Chr12qA3 no VAD (R) | TGGCTACTCCCAGAGGCTAA |
| **CRISPR gRNA primers** | |
| Chr9 break (F) | CAAGAGTGGTGCCCATACTCCTTG |
| Chr9 break (R) | GAGTATGGGCACCACTCTTGCAAG |
| Control break (F) | GCCAACACCGGCCGTTGTGCCTTG |
| Control break (R) | GCACAACGGCCGGTGTTGGCCAAG |
| **Taqman Probes (5’6-FAM, 3’ Iowa Black FQ)** | |
| MVM P4-P38 Taqman probe | GGAGGCAAGGTTGGTCACTA |
| Chr9 CRISPR break (9qE1) Taqman probe | CACACTCTCTCTCCATATGATTTCA |
| Mouse *Ercc3* Taqman probe | AAAGCTTGCACCCTGCTTTAGTGGCC |
| Human *Ercc3* Taqman probe | GGGAAAGGGACTGCTGTGTA |
