# Supplementary material for: Parvovirus minute virus of mice interacts with sites of cellular DNA damage to establish and amplify its lytic infection
Source: eLife. 2018 Jul 20;7:e37750. doi: 10.7554/eLife.37750 (PMC6095691; doi:10.7554/eLife.37750)
Supplement: Transparent reporting form [file elife-37750-transrepform.docx]

***eLife’s* transparent reporting form**

We encourage authors to provide detailed information *within their submission* to facilitate the interpretation and replication of experiments. Authors can upload supporting documentation to indicate the use of appropriate reporting guidelines for health-related research (see [EQUATOR Network](http://www.equator-network.org/%20)), life science research (see the [BioSharing Information Resource](https://biosharing.org/)), or the [ARRIVE guidelines](http://www.plosbiology.org/article/info:doi/10.1371/journal.pbio.1000412) for reporting work involving animal research. Where applicable, authors should refer to any relevant reporting standards documents in this form.

If you have any questions, please consult our Journal Policies and/or contact us: [editorial@elifesciences.org](mailto:editorial@elifesciences.org).

**Sample-size estimation**

- You should state whether an appropriate sample size was computed when the study was being designed
- You should state the statistical method of sample size computation and any required assumptions
- If no explicit power analysis was used, you should describe how you decided what sample (replicate) size (number) to use

Please outline where this information can be found within the submission (e.g., sections or figure legends), or explain why this information doesn’t apply to your submission:

No explicit power analysis was used in the sample size estimation while designing this study. This work was performed in cultured mammalian cells and not in primary samples. Conclusions were drawn from independent replicates of MVM infections which were subsequently processed for downstream analysis.

**Replicates**

- You should report how often each experiment was performed
- You should include a definition of biological versus technical replication
- The data obtained should be provided and sufficient information should be provided to indicate the number of independent biological and/or technical replicates
- If you encountered any outliers, you should describe how these were handled
- Criteria for exclusion/inclusion of data should be clearly stated
- High-throughput sequence data should be uploaded before submission, with a private link for reviewers provided (these are available from both GEO and ArrayExpress)

Please outline where this information can be found within the submission (e.g., sections or figure legends), or explain why this information doesn’t apply to your submission:

High throughput sequencing assays (including ChIP-seq and V3C-seq) were performed in at least two independent biological replicates of MVM infections according to ENCODE recommendations (Landt et al., *Genome Research*, 2012). “Independent biological replicates” refer to independently cultured cells that were synchronized, infected and processed for analyses. All high throughput data has been uploaded to the GEO server under the accession code **GSE112957**. The private token reviewers may use to access the data while it is in private status is: **srufuwisdhiplgf**. Further information can be found in the Materials and methods section of the manuscript. The second replicate of gamma-H2AX at 20 hours post infection yielded few peaks, and was inconsistent with the findings of high cellular levels of gamma H2AX at 20 hours assayed by western blot and the presence of many damaged sites by immunofluorescense assays. Therefore, the second replicate of gammaH2AX at 20 hours was not included in the downstream analysis. Focused imaging, qPCR and western blot analyses were performed in at least three independent replicates. Further information on the number of experimental replicates can be found in the respective figure legends and in the materials and methods section.

**Statistical reporting**

- Statistical analysis methods should be described and justified
- Raw data should be presented in figures whenever informative to do so (typically when N per group is less than 10)
- For each experiment, you should identify the statistical tests used, exact values of N, definitions of center, methods of multiple test correction, and dispersion and precision measures (e.g., mean, median, SD, SEM, confidence intervals; and, for the major substantive results, a measure of effect size (e.g., Pearson's r, Cohen's d)
- Report exact p-values wherever possible alongside the summary statistics and 95% confidence intervals. These should be reported for all key questions and not only when the p-value is less than 0.05.

Please outline where this information can be found within the submission (e.g., sections or figure legends), or explain why this information doesn’t apply to your submission:

Descriptions of statistical tests have been provided in the figure legends accompanying the relevant datasets.

(For large datasets, or papers with a very large number of statistical tests, you may upload a single table file with tests, Ns, etc., with reference to sections in the manuscript.)

**Group allocation**

- Indicate how samples were allocated into experimental groups (in the case of clinical studies, please specify allocation to treatment method); if randomization was used, please also state if restricted randomization was applied
- Indicate if masking was used during group allocation, data collection and/or data analysis

Please outline where this information can be found within the submission (e.g., sections or figure legends), or explain why this information doesn’t apply to your submission:

Samples were allocated into experimental groups according to their treatment status with DNA damaging agent, mock treatment or the number of hours of MVM infection. The treatment was validated using western blots for DNA damage markers, MVM-NS1 and Southern blots for replicating viral genome.

**Additional data files (“source data”)**

- We encourage you to upload relevant additional data files, such as numerical data that are represented as a graph in a figure, or as a summary table
- Where provided, these should be in the most useful format, and they can be uploaded as “Source data” files linked to a main figure or table
- Include model definition files including the full list of parameters used
- Include code used for data analysis (e.g., R, MatLab)
- Avoid stating that data files are “available upon request”

Please indicate the figures or tables for which source data files have been provided:

Source data for high-throughput experiments, including V3C-seq and ChIP-seq (presented in Figs 2, 3, 4) have been uploaded to the GEO repository, and can be accessed under the accession code **GSE112957**. The private token reviewers may use to access the data while it is in private status is: **srufuwisdhiplgf**. All bioinformatics code used for analysis have been provided in the Materials and Methods section of the manuscript.
